# Point of care ultrasound competency in recent medical school graduates: what to expect from trainees when designing an ultrasound curriculum

**DOI:** 10.1186/s12909-026-08878-5

**Published:** 2026-02-24

**Authors:** Alex Ryden, Aaron Crosby, Brian Poole, Richard Rose, Colton Long, David Haak, Caroline Milne

**Affiliations:** 1 Department of Medicine, Spencer Fox Eccles School of Medicine, University of Utah, Utah (30 North Mario Capecchi Dr, 3rd floor North, Salt Lake City, UT 84112 USA; 2 Department of Veterans Affairs, George E Wahlen VA Medical Center, Utah (500 Foothill Blvd, Salt Lake City, UT 84148 USA; 3 Division of Pulmonary Medicine, Spencer Fox Eccles School of Medicine, University of Utah, Utah (30 N Mario Capecchi Dr, 2nd Floor North Salt Lake City, Salt Lake City, UT 84112 USA

**Keywords:** education, residency, curriculum, medical school, internal medicine, pocus

## Abstract

**Introduction:**

Integration of Point-of-care ultrasound (POCUS) training into residency curricula has been pursued in a variety of specialties. There are no standard milestones for medical student POCUS education or competency. We designed a cross-sectional, single-center study of incoming recent medical school graduates at our Internal Medicine residency program to assess POCUS exposure, knowledge and aptitude.

**Methods:**

In 2023, we assessed the POCUS training, knowledge and skill of incoming PGY-1 internal medicine residents at a single center via a novel survey, written knowledge assessment, and an Observed Structured Clinical Exam (OSCE) developed for this study. The OSCE measured POCUS image acquisition skill for the following exams: focused cardiac, inferior vena cava, internal jugular, pulmonary, and renal. The results were analyzed via univariate analysis to identify potential associations between medical school POCUS experiences and subsequent POCUS knowledge and skill.

**Results:**

Our study included 61 of 61 incoming PGY-1 internal medicine trainees representing 45 different medical schools. While 80% of participants received ultrasound training in medical school, OSCE performance was variable. Those who received medical school POCUS training obtained an average of 2.71 out of a possible 5.00 acceptable images on the OSCE, while those who were untrained were able to obtain 1.77 out of a possible 5.00 (difference = 0.94, p=0.029). A variety of specific medical school experiences were associated with improved OSCE performance, including preclinical didactic sessions, POCUS use on clinical rotations, and POCUS use on sub-Internship rotations. POCUS use during sub-internship rotations was the only aspect of training associated with improved knowledge assessment performance.

**Conclusions:**

While 80% of participants reported some form of POCUS training in medical school, the setting, manner, and content of ultrasound training was variable, as was performance on assessments of POCUS knowledge and skill. Image acquisition skill was generally higher among those who received hands on training in medical school, but even within this group performance was highly variable. While this single-center, cross sectional study should be interpreted with caution, it is apparent that residency programs designing POCUS curricula should account for variation in baseline POCUS competency in recent medical school graduates.

**Supplementary Information:**

The online version contains supplementary material available at 10.1186/s12909-026-08878-5.

## Introduction

Point-of-care ultrasound (POCUS) is an important tool for modern clinician. Medical schools and residency programs in many different specialties are placing an emphasis on education in proper image acquisition and interpretation. In a 2020 survey of 154 MD-granting medical schools, 57% of respondents reported having a formal ultrasound curriculum [1]. For the purposes of this study, we considered any intentional, structured inclusion of POCUS training in didactics, skills training, or clinical practice as constituting a formal curriculum, whereas incidental exposure to POCUS technique and teaching from preceptors in the course of clinical rotations represented more informal training. In general, any mention of training or exposure to specific ultrasound skills or concepts in this paper is meant to be inclusive of both formal and informal teaching.

POCUS training is a required element for post-graduate training in some specialties, including family medicine and emergency medicine. While not required for internal medicine, the Alliance of Academic Internal Medicine and the Canadian Internal Medicine Interest Group recommend including POCUS education in residency [2, 3]. Most medical schools report that POCUS training occurs during the pre-clinical years – a time when student experiences have been shown to be variable across curriculums [4, 5, 6]. POCUS expertise among medical school faculty also varies, exacerbating variability in medical student learning [1, 7].

Milestones for medical student POCUS education are not yet standardized [8]. There is no guidance for integrating the use of POCUS into students’ existing clinical skills [9]. Prior studies have not evaluated medical school POCUS curricula from the students’ perspective, which may differ from what medical schools formally report about training offered and may be more indicative of retained knowledge [10–13].

We sought to understand the nature of prior POCUS training among our incoming PGY-1 class, a heterogenous group of recent medical school graduates, to inform curriculum design for our program. We designed this single center, cross-sectional study to assess incoming interns’ baseline training, knowledge and skills in POCUS. Our interest in this study was to determine what specific POCUS experiences our residents were bringing from medical school and how this impacted their baseline knowledge and POCUS skill.

## Materials & methods

Our 2023 study included all 61 PGY-1 categorical (33 internal medicine, 3 medicine/pediatrics) and preliminary residents (25) in the PGY-1 class of AY 2023–2024 at the ACGME accredited University of Utah Internal Medicine Residency training program. 45 unique medical schools were represented in this class of residents.

We evaluated previous POCUS training, knowledge, and image acquisition skills of participants via survey, a written knowledge assessment, and an Observed Structured Clinical Exam (OSCE) featuring standardized patients. The survey and knowledge assessment were developed for this study, have not been previously published, and can be seen in Appendix 1 (see Supplementary File). Responses were gathered via REDCap^®^. Responses were anonymous and were linked to an anonymous identifier generated by the respondent that was then used to link their responses to subsequent assessments.

The survey asked residents about their exposure to POCUS training in medical school, including specific POCUS exams they were taught, perceived quality of training, and self-reported skill in each exam. The design of the survey was pragmatic. In addition to demographic information, we assessed participants’ recollection of the content and quality of their medical schools’ POCUS curriculum as this was felt to better capture their subjective educational experience than self-reported curriculum data from medical schools. Questions used a 5-point Likert scale when applicable (supplementary appendix). We developed and administered a novel 15-point knowledge assessment with items assessing the residents’ knowledge of ultrasound theory, image interpretation, and the application of POCUS to clinical vignettes (supplementary appendix). The knowledge assessment was developed by two ABIM Certified Internal Medicine physicians with formal ultrasound training by the American College of Physicians and extensive ultrasound experience, and was reviewed by an expert POCUS user with board certification in Pulmonary and Critical Care Medicine. The content of the questions mapped to 70% image interpretation, 20% ultrasound theory, and 10% clinical reasoning.

Participants also participated in an OSCE with standardized patients in which they were to obtain images in the following domains: focused cardiac, inferior vena cava (IVC), internal jugular vein (IJ), renal, and pulmonary ultrasound. The cardiac exam consisted of either a parasternal long axis or apical 4 chamber view. The pulmonary ultrasound focused on identifying lung sliding. The renal exam was judged to be adequate if either the left or right kidney could be clearly identified. These exams were chosen as representative of common images obtained in internal medicine POCUS use. A scoring rubric with descriptions of the Butterfly IQ + ™ presets used can be found in the supplementary material with examples of adequate and inadequate images. Residents were given a total of 5 min to perform the exams with the use of Butterfly IQ + ™ ultrasound probes. Preceptors selected the appropriate ultrasound setting for each exam in case the resident was not familiar with the Butterfly IQ + ™ interface (settings noted in scoring rubric in supplementary file). 5 faculty, all experienced POCUS users, rated the images acquired as being adequate or inadequate for clinical interpretation. Raters were blind to the training and educational background of study participants. A binary approach to scoring the images was selected as it was felt that partial competence in image acquisition was of little clinical value, and in some cases could be more dangerous than not knowing how to obtain images at all. Each image was graded by one faculty rater, so inter-rater agreement data is not available, but faculty preceptors reviewed images from standardized patients prior to the OSCE to create agreement on acceptable and unacceptable images. All standardized patients who participated in the OSCE were examined by the faculty preceptors and determined to have adequate ultrasonic windows for POCUS exams performed.

Data analysis was performed in Microsoft^®^ Excel^®^. Univariate analysis was performed. Categorical variables were compared using Fisher’s exact test with statistical significance defined as *p* < 0.05. Continuous variables were compared using a two-tailed t-test, assuming unequal variances, with statistical significance defined as *p* < 0.05.

The Institutional Review Board of the University of Utah School of Medicine reviewed and provided approval for this study as part of our program’s umbrella IRB to study educational initiatives, and all participants provided informed consent for this specific study. All data was de-identified and labeled using an anonymous identifier.

## Results

**Fig. 1:**
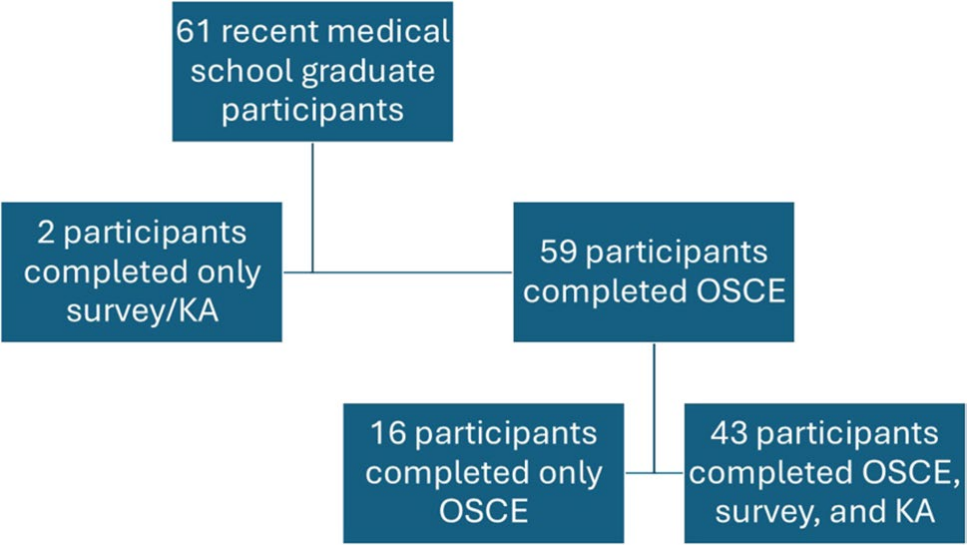
Flowchart of study participants. Participants who completed only the OSCE, knowledge assessment, or survey components of the study were excluded from analyses involving the portions of the study they did not complete

61 of 61 (100%) eligible residents consented for enrollment and participated in at least part of the assessments. Completion rates for various elements of the study are summarized in the flowchart in Fig. 1. 45/61 (73.8%) residents completed the survey and knowledge assessment (KA) and 59/61 (96.7%) residents completed the Objective Structured Clinical Examination (OSCE). 43/61 (70.5%) residents completed both the survey/KA and OSCE, 2/61 (3.3%) residents completed the survey/KA alone, and 16/61 (26.2%) residents completed only the OSCE. Residents who completed only the OSCE, knowledge assessment, or survey components of the study were excluded from analyses involving the portions of the study they did not complete.

Among residents who completed the POCUS survey, 73% (*n* = 33/45) indicated that they planned on pursuing a career that uses POCUS. 40% of residents (*n* = 18/45) reported that their medical schools had a formal POCUS curriculum, and of the remainder, 67% of residents (*n* = 18/27) reported receiving informal training during their clinical rotations. When queried about their medical school POCUS training experiences, 60% (*n* = 27/45) of residents reported receiving preclinical didactic sessions, 40% of residents (*n* = 18/45) participated in didactic sessions during clinical rotations, 62% (*n* = 28/45) received training on their core clinical rotations, 36% (*n* = 16/45) on sub-internships, and 33% (*n* = 15/45) took a POCUS elective. In terms of hands-on training, 44% of residents (*n* = 20/45) spent time in a POCUS simulation lab, 40% (*n* = 18/45) had the opportunity to practice scanning standardized patients, 62% (*n* = 28/45) scanned patients as part of their inpatient clinical rotations, and 18% (*n* = 8/45) performed POCUS exams on patients as part of their outpatient rotations. Regarding the quality of their medical school POCUS training, 80% (*n* = 36/45) of residents answered. Of these, 39% (*n* = 14/36) rated their training as very good or good, and 19% (*n* = 7/36) as bad. Similarly, 36% (*n* = 13/36) of residents reported being very satisfied or satisfied with their POCUS training, with 22% (*n* = 8/36) reporting being dissatisfied with their training. No residents indicated their training was very bad or reported being very dissatisfied.

Regarding the content of medical school POCUS training, 62% (*n* = 28/45) of residents reported focused Cardiac POCUS training, 49% (*n* = 22/45) Lung, 49% Liver (*n* = 22/45), 44% Bladder (*n* = 20/45), 42% Renal (*n* = 19/45), 40% Gallbladder (*n* = 18/45), 33% Musculoskeletal (*n* = 15/45), and 16% Deep Venous Thrombosis (*n* = 7/45).

The 45 participants who completed the knowledge assessment (KA) recorded an average score of 10.0 out of a possible 15.0. We assessed the association between receiving various methods of POCUS training with KA performance. While POCUS training during an acting sub-Internship was associated with improved KA performance (11.07 items correct vs. 9.50 out of a potential 15, difference = 1.57, *p* = 0.05), participating in pre-clinical POCUS didactics (10.38 vs. 9.53, difference = 0.86, *p* = 0.302), clinical year didactics (10.35 vs. 9.85, difference = 0.51, *p* = 0.529), clinical rotation POCUS use (10.33 vs. 9.56, difference = 0.77, *p* = 0.328), and completion of an ultrasound elective (10.87 vs. 9.61, difference = 1.26, *p* = 0.144) were not associated with a significant improvement in KA scores.

Among all residents who completed the POCUS OSCE, 27% (*n* = 16/59) were able to acquire a satisfactory cardiac view, 10% (*n* = 6/59) were able to obtain an adequate inferior vena cava (IVC) image, 68% (*n* = 40/59) were able to find and identify the internal jugular (IJ), 49% (*n* = 29/59) were able to demonstrate lung sliding, and 58% (*n* = 34/59) were able to show an image of a kidney that was adequate for interpretation.

We compared image acquisition between residents with and without specific training in POCUS for the relevant organ system. Associations were determined using a two-tailed Fisher’s exact test (Table 1). Those with exam specific training were significantly more proficient at the cardiac (44.4% satisfactory images vs. 12.5%, OR 5.6, *p* = 0.044), pulmonary (76.2% vs. 22.7%, OR 10.9, *p* = 0.0007), and renal exams (73.7% vs. 37.5%, OR 4.67, *p* = 0.031). No association was identified with receiving training and being able to obtain an acceptable image of the IVC (18.5% vs. 0%, OR ∞, *p* = 0.13), or the IJ (68.0% vs. 66.7%, OR 1.06, *p* = 1.0).

**Table 1:** POCUS OSCE Performance by Location and Modality of Medical School POCUS Training

		Average Images Acquired out of 5			
		Yes	No	Difference	*p*-value
	Any Med School Curriculum	2.71	1.77	0.94	0.029
Location of training	Preclinical Didactics	2.54	1.53	1.01	0.015
	Clinical Year Didactics	2.47	1.92	0.55	0.221
	Core Clinical Rotation Use	2.52	1.50	1.02	0.009
	Sub-I Rotation Use	2.80	1.79	1.01	0.024
	Ultrasound Elective	2.67	1.86	0.81	0.089
Modality of Training	Sim Lab	2.42	1.92	0.50	0.246
	Standardized Patients	2.71	1.77	0.94	0.023
	Inpatients	2.52	1.50	1.02	0.008
	Outpatients	3.00	1.94	1.06	0.019

Out of the five images each resident was asked to obtain during the OSCE, an average number of satisfactory images obtained was calculated, and these averages were compared between interns who did and did not receive medical school POCUS training using two-sample t-tests assuming unequal variance (Table 2). Residents with medical school POCUS training were able to obtain more images on average than those whose school had no formal curriculum (2.71 satisfactory images out of a possible 5 vs. 1.77, difference = 0.94, *p* = 0.029). Training during various phases of the medical curriculum was found to be associated with improved image acquisition during the OSCE, including preclinical didactic sessions (2.54 satisfactory images vs. 1.53, difference = 1.01, *p* = 0.015), use on clinical rotations (2.52 vs. 1.5, difference = 1.02, *p* = 0.009), and use on sub-Internship rotations (2.80 vs. 1.79, difference = 1.01, *p* = 0.024). Didactic sessions during the clinical years (2.47 images vs. 1.92, difference = 0.55, *p* = 0.55) and the completion of an ultrasound elective (2.67 images vs. 1.86, difference = 0.81, *p* = 0.089) were not associated with improved OSCE performance.

**Table 2:** POCUS Knowledge Assessment Performance by Location and Modality of Medical School POCUS Training

		Assessment Score out of 15			
		Yes	No	Diff	*p*-value
	Any Med School Curriculum	10.82	9.54	1.28	0.112
Location of training	Preclinical Didactics	10.38	9.53	0.86	0.302
	Clinical Year Didactics	10.35	9.85	0.51	0.529
	Core Clinical Rotation Use	10.33	9.56	0.77	0.328
	Sub-I Rotation Use	11.07	9.50	1.57	0.050
	Ultrasound Elective	10.87	9.61	1.26	0.144
Modality of Training	Sim Lab	10.11	10	0.11	0.890
	Standardized Patients	10.76	9.58	1.18	0.140
	Inpatients	10.44	9.38	1.06	0.187
	Outpatients	10.14	10	0.14	0.872

We assessed OSCE performance for residents trained using different modalities. Training using standardized patients (2.71 satisfactory images out of a possible 5 vs. 1.77, difference 0.94, *p* = 0.023), real-world use on hospitalized patients (2.52 images vs. 1.50, difference = 1.02, *p* = 0.008), and real-world use on outpatients (3.00 images vs. 1.94, difference = 1.06, *p* = 0.019) were all associated with improved OSCE performance, while the use of simulation training with virtual ultrasound trainers was not (2.42 images vs. 1.92, difference = 0.50, *p* = 0.246).

## Discussion

In keeping with previous studies, most incoming internal medicine residents in this study reported considering POCUS training to be important for their careers [14].

While 80% of our residents reported some form of ultrasound training, whether formal or informal, during medical school, the setting, manner, and content of ultrasound training was highly variable with training in settings that varied from pre-clinical didactic sessions to ultrasound-centered electives. Hands-on training methodologies also greatly varied. Though the focused cardiac exam was the most frequently covered POCUS exam in medical school curricula with 62% of incoming PGY-1s reporting its inclusion, training in no other organ system POCUS exam was reported by more than 50% of residents. It is not surprising that residents had highly variable perceptions of the quality of their POCUS training, with less than half reporting being Satisfied or Very Satisfied with their medical school’s POCUS curriculum.

Despite the focus on the cardiac POCUS examination in undergraduate medical curricula, only 27% of recent medical school graduates were able to obtain a single cardiac view of the heart, and only 10% were able to obtain an acceptable view of the inferior vena cava. While receiving medical school training in cardiac POCUS was associated with improved performance in obtaining a cardiac view, even students with cardiac POCUS training were only able to obtain acceptable image 44.4% of the time. Performance in the IVC exam, which is often part of instruction in the focused cardiac exam, was very poor in both the trained and untrained cohorts.

In contrast, performance on the pulmonary and renal POCUS exams was much more favorable than for the cardiac exam, with trained participants again outperforming the untrained. Finally, the majority of incoming PGY-1s were able to idenitfiy the internal jugular vein with no statistically significant difference between those who had received vascular or central line training and those who had not.

We administered a 15-point POCUS Knowledge Assessment (KA) to assess the knowledge of our residents regarding ultrasound theory, image interpretation, and integration of POCUS into clinical decision making. Overall, PGY-1s who had received the opportunity to use POCUS during their clinical sub-internship performed significantly better on the KA than those who had not, while other training settings and modalities, including having a formal ultrasound elective, were not associated with better KA performance. While this result may be due to the limited power of our single center study, it may reflect the value of integrating POCUS training into a setting such as the sub-internship where a student is tasked with making clinical decisions at a higher level than at any previous point in their medical training.

We examined the setting and modality of POCUS training and its association with performance on image acquisition ability in an OSCE using standardized patients. Pre-clinical POCUS didactic training, clinical-year hands-on training, and use of POCUS on a sub-internship rotation were associated with improved image acquisition performance, while clinical year didactic sessions and completion of an ultrasound elective were not. This may reflect hands-on practice for image acquisition being displaced by time spent in POCUS didactics during the clinical years. The percentage of residents who completed an ultrasound elective was small at 33% (*n* = 15/45), so the study may have been underpowered to identify an association between elective completion and OSCE performance.

Our study found an association between hands-on practice scanning patients in medical school and higher performance in obtaining images acceptable for interpretation on our OSCE. The use of simulation centers and virtual ultrasound trainers in medical school were not associated with improved OSCE performance in our study, though less than half (44%) of respondents had exposure to these facilities, so our study may have lacked the power to identify such an association .

Our study highlights several challenges and opportunities for educators in the field of point-of-care ultrasound. Trainees view POCUS to be an important skill they will need for their future practice, but the training received by medical students is variable. Faculty developing POCUS training for residents will need to consider that a large proportion of residents start residency with minimal POCUS background. For educators training medical students, our study suggests that hands-on practice with patients is strongly associated with image acquisition skill on subsequent assessment. In our cross-sectional study, the highest performance in knowledge and clinical application of POCUS was seen in residents who had POCUS integrated into their sub-internships. This argues that there may be a benefit to ensuring POCUS curricula remain grounded in evidence-based medicine and clinical reasoning.

Our study has some limitations. It was a cross-sectional study performed at a single site, and thus had a relatively small sample size of recent medical school graduates that may not be representative of all medical school graduates, though our sample did include graduates of 45 different medical schools. Our evaluation of the training that our residents received was based on their report rather than direct examination of the curricula of each medical school, though we feel that evaluating the curricula of schools via graduate report is representative of the students’ actual experience. The binary approach to scoring images as “acceptable” vs. “unacceptable” for interpretation does not capture partial competence as well as a Likert scale would, but we feel that from a pragmatic standpoint, in clinical practice images are either usable for clinical decision-making or they are not. Partial competence and the acquisition of a suboptimal image can be worse than not obtaining an image at all. Finally, our study is meant to be a starting point for hypothesis generation, and multivariable analysis using larger sample sizes from multiple sites would be necessary to draw more definitive conclusions.

The largest area of opportunity identified in our study is for the standardization of POCUS competencies and training. As it stands, POCUS skill and experience among medical school graduates remains variable. The optimal combination of POCUS competencies to expect from medical school graduates remains an area for further research and discussion, but we would advocate for POCUS training that emphasizes the teaching of image acquisition through hands-on practice and the integration of POCUS into the training of clinical reasoning.

## Conclusions

While we found that most incoming interns received some amount of ultrasound training in medical school, the majority had neutral or mildly negative opinions regarding the training they received. Image acquisition skill was generally higher among those who received hands on training in medical school, but was highly variable. The aspect of training most associated with improved performance on a written test of image interpretation and clinical reasoning was the use of POCUS during sub-internship rotations. Because we found baseline skills and knowledge regarding POCUS to be highly variable among recent medical school graduates, we recommend that GME leaders developing POCUS curricula begin with the basics and make sure all of their learners possess the fundamental skills and knowledge to progress to more advanced POCUS applications.

## Supplementary Information

Supplementary Material 1.

Supplementary Material 2.

## Data Availability

The datasets used and/or analyzed during the current study are available from the corresponding author on reasonable request.
