# Supplementary material for: Point of care ultrasound competency in recent medical school graduates: what to expect from trainees when designing an ultrasound curriculum
Source: BMC Med Educ. 2026 Feb 24;26:519. doi: 10.1186/s12909-026-08878-5 (PMC13037012; doi:10.1186/s12909-026-08878-5)
Supplement: Supplementary file 1 — Supplementary Material 1. [file 12909_2026_8878_MOESM1_ESM.pdf]

Intern Medical School Ultrasound Education Survey

Please complete the survey below.

Thank you!

Demographics

Please enter the first letter of your birth month (lower case) and the last 4 digits of your cellphone number: ________________________________

Please select if you are a categorical or preliminary intern:
- ◯ Categorical
- ◯ Med/Peds
- ◯ Prelim-Anesthesia
- ◯ Prelim-Dermatology
- ◯ Prelim-Neurology
- ◯ Prelim-Ophthalmology
- ◯ Prelim-PM&R

Please specify if MD or DO:
- ◯ MD
- ◯ DO

Do you currently plan on pursuing a career that utilizes bedside ultrasound in some capacity?
- ◯ Yes
- ◯ No
- ◯ Not sure

Do you currently plan to do a fellowship?
- ◯ Yes
- ◯ No

We will first ask questions about your exposure to ultrasound during medical school.

My medical school had a formal ultrasound curriculum:
- ◯ Yes
- ◯ No
- ◯ Unsure

My school did not have a curriculum but I received ultrasound instruction during medical school:
- ◯ Yes
- ◯ No

In what settings did you receive teaching on ultrasound? (select all that apply)
- ☐ Pre-Clinical
- ☐ Clinical Year Didactics
- ☐ Clinical Year On Rotation
- ☐ Sub-Internship
- ☐ Elective Rotation

What kind of ultrasound training did you recieve?
- ◯ Classroom
- ◯ Hands On
- ◯ Both

In regards to hands on ultrasound training, what scenarios were you exposed to ? (select all that apply)
- ☐ Sim Lab
- ☐ Standardized Patient
- ☐ Patients During an Inpatient Rotation
- ☐ Patients During an Outpatient Rotation

For classroom ultrasound training, how many hours of education do you estimate you recieved?

◯ 1-2 hours
◯ 3-5 hours
◯ 6-7 hours
◯ 8-10 hours
◯ > 10 hours

For hands on training, how many hours of education with the sim lab do you estimate you recieved?

◯ 1-2 hours
◯ 3-5 hours
◯ 6-7 hours
◯ 8-10 hours
◯ > 10 hours

For hands on training, how many standardized patients do you estimate you scanned?

◯ 1-2 patients
◯ 3-5 patients
◯ 6-7 patients
◯ 8-10 patients
◯ > 10 patients

For hands on training, how many patients in an inpatient setting do you estimate you scanned?

◯ 1-2 Patients
◯ 3-5 Patients
◯ 6-7 Patients
◯ 8-10 Patients
◯ > 10 Patients

For hands on training, how many patients in an outpatient setting do you estimate you scanned?

◯ 1-2 Patients
◯ 3-5 Patients
◯ 6-7 Patients
◯ 8-10 Patients
◯ > 10 Patients

How would you rate the quality of ultrasound training you received during medical school?

◯ Very Poor
◯ Poor
◯ Acceptable
◯ Good
◯ Very Good

How would you rate your satisfaction with the ultrasound training you recieved during medical school?

◯ Very Dissatisfied
◯ Dissatisfied
◯ Neither Satisfied nor Dissatisfied
◯ Satisfied
◯ Very Satisfied

Next, we will ask questions about specific ultrasound skills.

What fields of ultrasound did you receive ultrasound instruction on during medical school? (Select all that apply)

☐ Focused Cardiac Ultrasound
☐ Pulmonary Ultrasound
☐ Liver Ultrasound
☐ Gallbladder Ultrasound
☐ Renal Ultrasound
☐ Bladder Ultrasound
☐ Aortic Aneurysm Ultrasound
☐ Deep venous Thrombosis Ultrasound
☐ Musculoskeletal Ultrasound
☐ Other
☐ None of the Above

If you were trained on an ultrasound skill not described above, please enter it here: ______________________________

What procedural skills did you receive ultrasound instruction on during medical school? (select all that apply)

- ☐ Peripheral IV
- ☐ Central Line
- ☐ Arterial Line
- ☐ Thoracentesis
- ☐ Paracentesis
- ☐ Lumbar Puncture

Please rate the quality of ultrasound training you received on the following ultrasound skills:

	Very Poor	Poor	Acceptable	Good	Very Good
Focused Cardiac Ultrasound	○	○	○	○	○
Pulmonary Ultrasound	○	○	○	○	○
Liver Ultrasound	○	○	○	○	○
Gallbladder Ultrasound	○	○	○	○	○
Renal Ultrasound	○	○	○	○	○
Bladder Ultrasound	○	○	○	○	○
Aortic Aneurysm Ultrasound	○	○	○	○	○
Deep Venous Thrombosis Ultrasound	○	○	○	○	○
Musculoskeletal Ultrasound	○	○	○	○	○
Central Line Placement	○	○	○	○	○
Arterial Line Placement	○	○	○	○	○
Thoracentesis	○	○	○	○	○
Paracentesis	○	○	○	○	○
Lumbar Puncture	○	○	○	○	○

Please rate your ability in the following ultrasound skills:

	Very Poor	Poor	Acceptable	Good	Very Good
Focused Cardiac Ultrasound	○	○	○	○	○
Pulmonary Ultrasound	○	○	○	○	○
Liver Ultrasound	○	○	○	○	○
Gallbladder Ultrasound	○	○	○	○	○
Renal Ultrasound	○	○	○	○	○
Bladder Ultrasound	○	○	○	○	○
Aortic Aneurysm Ultrasound	○	○	○	○	○
Deep Venous Thrombosis Ultrasound	○	○	○	○	○
Musculoskeletal Ultrasound	○	○	○	○	○
Central Line Placement	○	○	○	○	○
Arterial Line Placement	○	○	○	○	○
Thoracentesis	○	○	○	○	○
Paracentesis	○	○	○	○	○
Lumbar Puncture	○	○	○	○	○

We will now ask a series of questions to evaluate your knowledge of ultrasound principles.

Use the image above for the following questions.

Using the above image, match the patient with the most appropriate probe for examination.

	Linear	Curvilinear	Phased Array
Patient presenting to the ED with chest pain, hypotension and hypoxia.	◯	◯	◯
Patient in clinic with swelling and pain of his left lower extremity.	◯	◯	◯
Patient with inability to urinate for 3 days.	◯	◯	◯

What view of the heart is shown in the above image?

◯ Four Chamber
◯ Parasternal Short Axis
◯ Parasternal Long Axis
◯ Subxiphoid

What valves are visualized in the above image?

☐ Aortic
☐ Mitral
☐ Tricuspid
☐ Pulmonic

What view of the heart is shown in the image above?

◯ Four Chamber
◯ Parasternal Short Axis
◯ Parasternal Long Axis
◯ Subxiphoid

What structure is shown in the circled area of the above image?

◯ Left Atrium
◯ Left Ventricle
◯ Right Atrium
◯ Right Ventricle
◯ None of the Above

What imaging finding is seen in the above image?

____

Patient presents with dyspnea and cough, you obtain the above ultrasounds. What intervention is indicated in this instance?

◯ antibiotics
◯ diuresis
◯ chest tube
◯ thoracentesis
◯ nebulizer therapy

In regards to the area of darkness in the above image, it would be described in ultrasound terminology as:

◯ Hypoechoic
◯ Hyperechoic
◯ Isoechoic
◯ None of the Above

What structure is shown in the above image?

◯ Gallbladder
◯ Spleen
◯ Liver
◯ Kidney
◯ Bladder
◯ None of the Above

In regards to the above image, is it normal or abnormal for that organ?

◯ Yes
◯ No
