# Supplementary figures and images for: Point of care ultrasound competency in recent medical school graduates: what to expect from trainees when designing an ultrasound curriculum
Source: BMC Med Educ. 2026 Feb 24;26:519. doi: 10.1186/s12909-026-08878-5 (PMC13037012; doi:10.1186/s12909-026-08878-5)

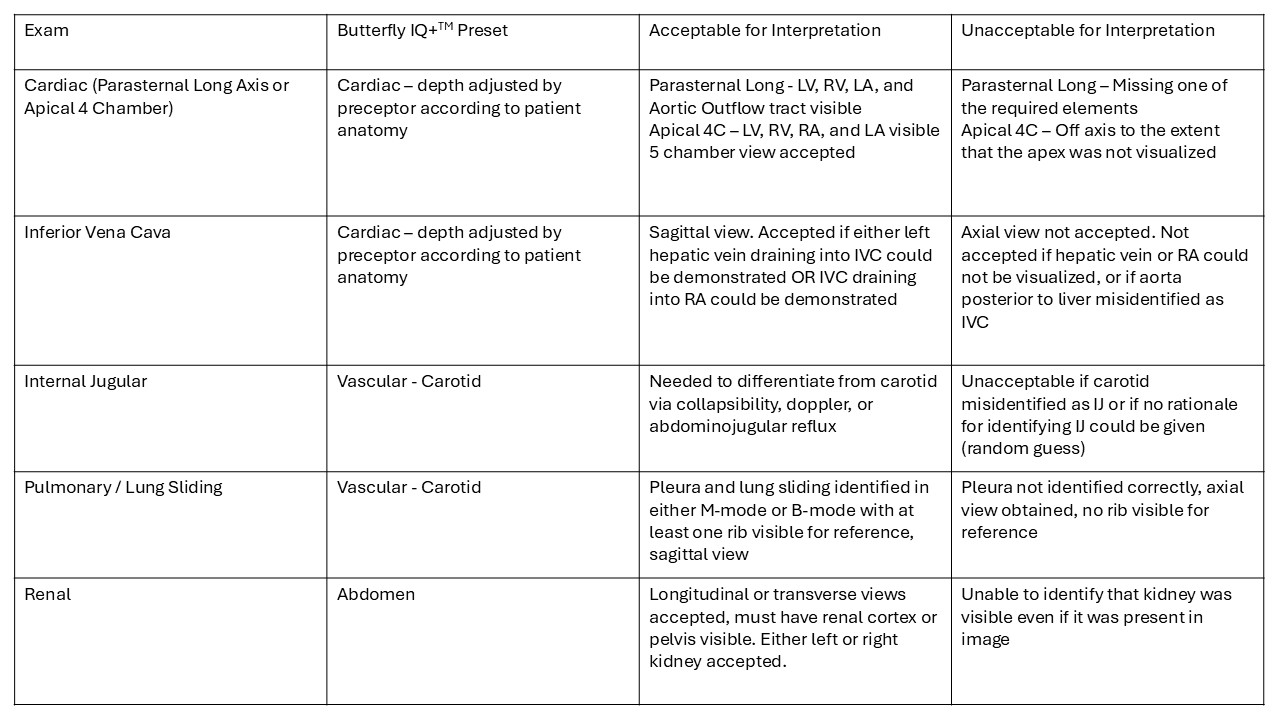

Supplement: Supplementary file 2 — Supplementary Material 2. [file 12909_2026_8878_MOESM2_ESM.jpg]
